# Digital Monitoring of Symptoms and Lung Function During Birch Pollen Season in Pediatric Patients

**DOI:** 10.1002/clt2.70101

**Published:** 2025-09-29

**Authors:** Tiina Helena Tanninen, Paula Hannele Reiterä, Annika Saarto, Janne Burman, Anna Susanna Pelkonen, Mika Juhani Mäkelä

**Affiliations:** ^1^ Department of Allergology University of Helsinki and Helsinki University Hospital Helsinki Finland; ^2^ Biostatistics Unit Department of Public Health University of Helsinki and Helsinki University Hospital Helsinki Finland; ^3^ Biodiversity Unit University of Turku Turku Finland

**Keywords:** allergic rhinitis, asthma, lung function, mHealth, pediatric

## Abstract

**Background:**

Mobile health (mHealth) applications for asthma and allergic rhinitis (AR) may guide patients in following medication use, symptoms, and lung function supporting self‐management.

**Objective:**

The primary study objective was to investigate the objective effect of birch pollen on asthma and AR symptoms and medicine use in pediatric patients with varying levels of birch‐specific immunoglobulin E (IgE) during the 2022 birch pollen season using digital tools. The secondary objectives were to determine the effect of birch pollen on Asthma Control Test scores, and to record the subjective benefits in self‐management while using the application.

**Methods:**

Altogether, 48 pediatric participants were categorized into three groups based on their birch‐specific IgE levels. Participants continued their existing asthma control therapy. For allergic rhinitis and conjunctivitis, antihistamines, intranasal corticosteroids (INCS) or a combination of INCS and intranasal antihistamines, and cromoglicates or local antihistamines were prescribed. The study involved daily asthma and allergic rhinitis symptom and medication reporting via the mHealth application (KAMU Health, Finland) combined with microspirometry during the birch pollen season in Helsinki, Finland.

**Results:**

The patients preferred oral AR treatment. However, the low birch pollen levels may have contributed to moderate adherence to AR treatment. A low birch pollen load does not significantly impair lung function in young patients receiving anti‐asthmatic treatment regularly. The majority of patients perceived this digital approach as beneficial, irrespective of their level of birch‐specific sensitization.

**Conclusion:**

Digital tools support asthma and AR care by enabling disease monitoring, patient engagement, and provide real‐world insights for clinicians.

## Graphical abstract

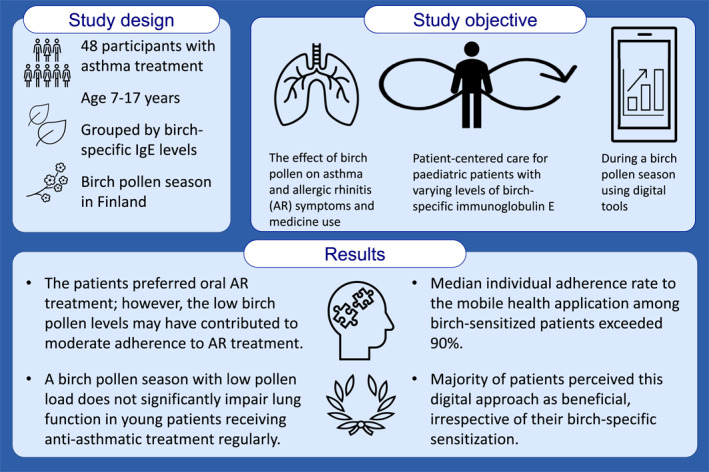

**Abbreviations**
ACT: Asthma Control Test
AR: allergic rhinitis
FEV1: forced expiratory volume in 1 second
IgE: immunoglobulin E
INAH: intranasal antihistamine
INCS: intranasal corticosteroid
mHealth: mobile health
OAH: oral antihistamine
SABA: short‐acting β‐agonist

To the Editor

During the transition from pediatric to adult care, mobile health (mHealth) applications for asthma and allergic rhinitis (AR) may guide patients in following medication use, symptoms, and lung function supporting self‐management [1]. Adherence to pharmacotherapy is often suboptimal during adolescence [2], as well as in patients with asthma [3] and AR [4]. Considering the variability of pollen exposure between pollen seasons and its impact on symptom control, especially among pollen sensitized individuals, digital tools offer a promising approach for monitoring.

The primary study objective of this pilot study was to investigate the objective effect of birch pollen on asthma and AR symptoms and medicine use in pediatric patients with varying levels of birch‐specific immunoglobulin E (IgE) during the 2022 birch pollen season [5] using digital tools. The secondary objectives were to determine the effect of birch pollen on Asthma Control Test (ACT) scores [6, 7] and SABA use, and to record the subjective benefits in self‐management while using the application. The participants were categorized into three groups based on their birch‐specific IgE levels (< 0.35 kU/L defining controls; 0.35 to < 17.5 kU/L, ≥ 17.5 kU/L). Participants continued their existing asthma control therapy throughout the study and took a short‐acting β‐agonist (SABA) as needed. For allergic rhinitis, intranasal corticosteroids (INCS) or a combination of INCS and intranasal antihistamines (INAH); and for conjunctivitis, cromoglicates or local antihistamines were prescribed. This study was conducted in 2022 at the Skin and Allergy Hospital, Helsinki University Hospital, Finland. The study involved a baseline visit, a follow‐up call after the birch pollen season, and daily asthma and allergic rhinitis symptom and medication reporting via the mHealth application (KAMU Health, Finland) combined with microspirometry during the birch pollen season. Analyses were conducted using IBM SPSS software (version 29, IBM Corp., Armonk, NY). The Helsinki University Hospital ethics committee approved the study (approval no. HUS/412/2022). The study was registered at The Helsinki University Hospital (registration no. HUS/458/2022). Written informed consent was signed by the participant and the parent (for participants aged < 15 years), according to Finnish legislation.

A total of 48 patients with anti‐inflammatory asthma treatment completed the study. Baseline characteristics of the participants are presented in Table 1. The mean age was 12 years (range 7–17 years). The median pediatric ACT score [6] for participants aged < 12 years was 24 and median ACT score [7] for participants aged ≥ 12 years was 23 at baseline.

**TABLE 1:** Baseline characteristics.

Baseline characteristics	Birch‐IgE < 0.35 kU/L (mean and 95% CI or median^a^ and IQR) [min; max] *n* = 16	Birch‐IgE 0.35 to < 17.5 kU/L (mean and 95% CI or median and IQR^a^) [min; max] *n* = 15	Birch‐IgE ≥ 17.5 kU/L (mean and 95% CI or median and IQR^a^) [min; max] *n* = 17	All participants (mean and 95% CI or median and IQR^a^) [min; max] *n* = 48	*p* value
Age, years, mean (95% CI)	12.1 (10.2–13.9) [7; 17]	11.9 (10.5–13.3) [7; 17]	10.8 (9.7–11.8) [8; 15]	11.6 (10.8–12.4) [7; 17]	0.341^b^
Gender, male, *n* (%)	8 (50.0)	10 (66.7)	10 (58.8)	28 (58.3)	0.642^d^
Birch‐specific IgE^a^, median (IQR)	0.2 (0.1–0.2) [0.01; 0.3]	3.4 (2.4–9.7) [0.4; 12.2]	77.6 (32.0–100.0) [17.7; 100.0]	3.4 (0.2–45.4) [0.01; 100]	**<** **0.001** ^c^ Neg versus low: **0.006** Low versus high: **0.004** Neg versus high: **<** **0.001**
Participants sensitized at least to one other allergen except the birch (timothy, mugwort, cat, dog, horse, *Cladosporium herbarum*, or *Dermatophagoides pteronyssinus*), *n* (%)	7 (44)	12 (80)	16 (94)	35 (73)	Neg versus low: 0.116 Low versus high: 0.687 Neg versus high: **0.005** ^e^
Medication				*n* = 48	
SABA used in last month, times^a^, median (IQR)	2 (0–5) [0; 30]	0 (0–1) [0; 10]	0 (0–2) [0; 30]	0 (0–2) [0; 30]	0.050^c^
GINA treatment classification					
GINA 2, *n* (%)	9 (56)	7 (47)	7 (41)	23 (48)	Neg versus low: 1.000 Low versus high: 1.000 Neg versus high: 1.000^e^
GINA 3, *n* (%)	2 (13)	5 (33)	7 (41)	14 (29)	Neg versus low: 0.552 Low versus high: 1.000 Neg versus high: 0.215^e^
GINA 4, *n* (%)	5 (31)	3 (20)	3 (18)	11 (23)	Neg versus low: 1.000 Low versus high: 1.000 Neg versus high: 1.000^e^
Lung function measured by spirometry at the hospital	*n* = 14	*n* = 14	*n* = 16	*n* = 44	
FVC *z* score (SD), mean (95% CI)	−0.7 (−1.4 to −0.0) [−2.8; 1.1]	−0.5 (−1.0 to −0.1) [−1.9; 0.9]	−0.9 (−1.5 to −0.3) [−3.5; 0.7]	−0.7 (−1.0 to −0.4) [−3.5; 1.1]	0.612^b^
FEV1 *z* score (SD), mean (95% CI)	−1.1 (−1.7 to −0.5) [−2.4; 0.6]	−1.3 (−1.8 to −0.8) [−3.0; 0.2]	−1.3 (−1.7 to −0.8) [−2.9; 0.3]	−1.2 (−1.5 to −0.9) [−3.0; 0.6]	0.843^b^
FEV1/FVC *z* score (SD), mean (95% CI)	−0.8 (−1.6 to 0.0) [−3.4; 1.5]	−1.3 (−2.0 to −0.7] [−3.2; 0.6]	−0.68 (−1.3 to −0.0) [−2.6; 1.4]	−0.9 (−1.3 to −0.5) [−3.4; 1.5]	0.310^b^
Asthma‐related symptoms				*n* = 48	
	*n* = 8	*n* = 6	*n* = 11	*n* = 25	
ACT, < 12 years old^a^, median (IQR)	24.5 (22.3 to 26.0) [21; 27]	25.0 (24.5 to 26.3) [23; 27]	24.0 (23.0; 24.0) [13; 25]	24.0 (23.0 to 25.0) [13; 27]	0.101^c^
	*n* = 8	*n* = 9	*n* = 6	*n* = 23	
ACT, ≥ 12 years old^a^, median (IQR)	21.5 (20.0 to 23.8) [17; 25]	25.0 (20.5 to 25.0) [18; 25]	22.5 (21.0 to 23.3) [21; 24]	23.0 (21.0 to 25.0) [17; 25]	0.342^c^
				*n* = 48	
Continuous cough, *n* (%)	2 (12.5)	0 (0.0)	3 (17.6)	5 (10.4)	0.348^d^
Nighttime cough, *n* (%)	2 (12.5)	0 (0.0)	1 (5.9)	3 (6.3)	0.638^d^
Light exercise‐induced breathing symptoms, *n* (%)	0 (0.0)	1 (6.7)	1 (5.9)	2 (4.2)	0.759^d^
Heavy exercise‐induced breathing symptoms, *n* (%)	8 (50.0)	5 (33.3)	12 (70.6)	25 (52.1)	0.107^d^
Symptoms in birch‐pollen season in 2021					
VAS for ocular symptoms, median (IQR)	0 (0 to 4)	3 (0 to 5)	5 (2 to 8)	3 (0 to 6)	**0.023** ^c^ Neg versus low: 1.000 Low versus high: 0.273 Neg versus high: **0.020**
VAS for nasal symptoms (IQR)	2.5 (0 to 5)	4 (0 to 6)	6 (4.5 to 7)	4.5 (2 to 6)	**0.002** ^c^ Neg versus low: 0.879 Low versus high: 0.070 Neg versus high: **0.002**

*Note:* Significant *p* values are shown in bold.

Abbreviations: 95% CI = 95% confidence interval, ACT = Asthma Control Test, FEV1 = forced expiratory volume in 1 second, FVC = forced vital capacity, GINA = Global Initiative for Asthma, ICS = inhaled corticosteroid, IgE = immunoglobulin E, IQR = interquartile range, LABA = long‐acting β‐agonist, SABA = short‐acting β‐agonist, SD = standard deviation, VAS = Visual Analog Scale (0 = no symptoms, 10 = severe symptoms).

^a^ Median.

^b^ One‐way ANOVA.

^c^ Independent‐samples Kruskal–Wallis test.

^d^ *χ* ^2^ test.

^e^ 2‐sample *Z*‐test, adjusted with the Bonferroni correction.

In 2022, the birch pollen season started on 30 April and ended on 25 May in Helsinki, as defined by The European Academy of Allergy and Clinical Immunology [5], with a daily maximum of 1018 birch pollen grains/m^3^. The total sum of the daily birch pollen was lower compared to the year before (> 6400 vs. > 66,000 grains/m^3^ in 2021; data provided by the University of Turku).

Due to moderate subjective need for symptomatic AR medication during the mild birch pollen season in 2022, the reported INCS use was generally limited. The percentage of adherent patients (percentage of days covered > 80%) during the birch pollen season was 7% and 12% for INCS, and 80% and 65% for anti‐asthmatic treatment, respectively, in the groups with low and high IgE levels for birch, without any significant differences between the groups. The median percentage of days the patients took oral antihistamines (OAH) was 62% and 73% in participants with low and high birch‐specific IgE levels, respectively. No statistically significant correlations were observed between FEV1, individual deviations in daily FEV1 performance or modified symptom load index and daily birch pollen concentration in any group. Median pediatric ACT scores did not differ between groups (24, 25, 25 in ascending group order) after the birch pollen season. However, the median ACT scores in patients aged ≥ 12 years were significantly higher in patients with low birch‐specific IgE levels compared to controls (25 vs. 22, *p* = 0.048). Almost two‐thirds of the participants did not need SABA during the birch pollen season, without any significant differences between the groups. Both groups of birch‐sensitized patients had significantly better median adherence at the individual level to the application than controls (92% vs. 54%, *p* = 0.009 and *p* = 0.011, respectively) (Figure 1). Seventy‐three percent of the participants reported subjective benefits in self‐management while using the application, without any significant differences between groups.

**FIGURE 1:**
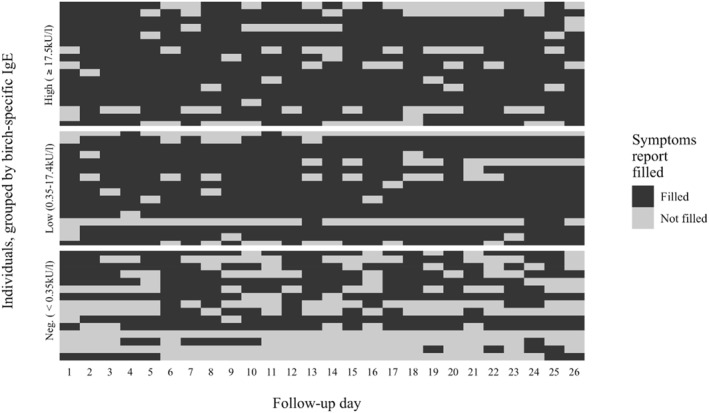
Individual adherence to application use. IgE = immunoglobulin E.

Participants with AR were advised to start INCS before and continue through the pollen season, supplementing with OAH and local treatments as needed. The increase in dust following snowmelt can cause mucosal irritation even in controls, potentially explaining the self‐administration of antihistamines. The low birch pollen levels may have contributed to moderate adherence to AR treatment. A birch pollen season with low pollen load does not significantly impair lung function in young patients receiving anti‐asthmatic treatment regularly, being in line with previous Scandinavian studies [8, 9]. The median individual adherence rate to the application among birch‐sensitized participants exceeded 90%. Our participants reported subjective benefits of the application for self‐management regardless of the level of birch‐specific sensitization.

A strength of this study is our response to the unmet need for pediatric studies that combine digital monitoring with asthma and AR management. The findings of this study are of local and clinical relevance, considering the high prevalence of pediatric asthma and the increasing intensity of birch pollen seasons in Finland due to climate change. The limitations of this study include the lack of microspirometry reproduction and validation of the application, the restricted amount of birch pollen in 2022 in Finland, and the limited number of participants.

In conclusion, the majority of patients perceived this digital approach as beneficial, irrespective of their level of birch‐specific sensitization. It demonstrates potential to improve disease monitoring and patient engagement in asthma and AR, while providing clinicians with valuable real‐world insights into symptoms and medication use.

## Author Contributions

**Tiina Helena Tanninen:** conceptualization, investigation, data curation, project administration, formal analysis, software, writing – review and editing, visualization, validation, methodology, writing – original draft, funding acquisition. **Paula Hannele Reiterä:** methodology, visualization, writing – review and editing, formal analysis, software. **Annika Saarto:** methodology, writing – review and editing, investigation. **Janne Burman:** investigation, writing – review and editing. **Anna Susanna Pelkonen:** conceptualization, investigation, methodology, validation, writing – review and editing, supervision. **Mika Juhani Mäkelä:** conceptualization, investigation, funding acquisition, methodology, validation, writing – review and editing, project administration, supervision, resources.

## Conflicts of Interest

The authors declare no conflicts of interest.

## Data Availability

The data that support the findings of this study are available from the corresponding author upon reasonable request.
